# Supplementary material for: Mental Health of Chinese Online Networkers under COVID-19: A Sociological Analysis of Survey Data
Source: Int J Environ Res Public Health. 2020 Nov 28;17(23):8843. doi: 10.3390/ijerph17238843 (PMC7730967; doi:10.3390/ijerph17238843)
Supplement: Supplementary file 1 [file ijerph-17-08843-s001.pdf]

Supplementary Material

Mental Health of Chinese Online Networkers Under COVID-19: A Sociological Analysis of Survey Data

Yang Xiao [1], Yanjie Bian [2,3,*] and Lei Zhang [4]

[1] School of Philosophy and Government, Shaanxi Normal University, Xi'an 710119, China; xiaoyang2017@snnu.edu.cn
[2] Institute for Empirical Social Science Research, Xi'an Jiaotong University, Xi'an 710049, China
[3] Department of Sociology, University of Minnesota, Minneapolis, MN 55455, USA
[4] Department of Sociology, University of Colorado Colorado Springs, Colorado Springs, CO 80918, USA; lzhang4@uccs.edu
* Correspondence: bianx001@umn.edu

Received: 28 October 2020; Accepted: 24 November 2020; Published: date

Supplementary 1. ANOVA and Alternative Methodologies in Reference to Figure 1 and Table 3

The classic ANOVA is sensitive to the violation of homoscedastic assumption (Moder, 2016) and normality assumption (Blanca et al. 2017). Accordingly, we obtain three sets of results about between-group-difference comparisons from procedures using different statistical assumptions as shown in Supplementary Table S1:

(1) The classic F-test-based ANOVA, under the homoscedastic assumption. Levene's test demonstrates that our results indeed violate this assumption.
(2) Welch's ANOVA (Welch, 1951), under the assumption of normality but without requiring equal variances of a dependent variable across test groups. As Supplementary Table S1 shows, the Welch's ANOVA results are also significant. Given the violation of homoscedastic assumption, we perform both Scheffe's tests and tests of Games-Howell (1976), as detailed in Supplementary Table S2 and briefly reported in Figure 1.
(3) Kruskal-Wallis nonparametric test of equality-of-population (Kruskal & Wallis, 1952), does not require normality of a dependent variable. This test is implemented by the "*kwallis*" command in Stata (StataCorp. 2015. *Stata 14 Base Reference Manual*. Stata Press: College Station, TX, USA.). It requires that the distributions of a dependent variable across test groups have a similar shape. Supplementary Figure S1 shows that our dependent variables satisfy this requirement. Therefore, we present the Kruskal-Wallis test results in Supplementary Table S1 and Figure 1.

Supplementary Table S1. Assumptions and Results from Different ANOVA Procedures.

Methods	Key Assumptions		Dependent Variables	Levene's Tests		ANOVA	
	Normality	Homogeneity of Variance		Test Scores	*p*-Values	Test Scores	*p*-Values
Classic ANOVA F-test	×	×	Depression	14.40	<0.001	17.20	<0.001
			Anxiety	25.39	<0.001	32.38	<0.001
			Somatization	54.19	<0.001	44.42	<0.001
Welch ANOVA	×		Depression			14.33	<0.001
			Anxiety			26.54	<0.001
			Somatization			31.22	<0.001
Kruskal-Wallis nonparametric ANOVA		Distributions of dependent variables across test groups share a similar shape.	Depression			34.34	<0.001
			Anxiety			65.36	<0.001
			Somatization			66.74	<0.001

Note:× means an assumption is needed for an ANOVA procedure.

Supplementary Table S2. Scheffe and Games-Howell ANOVA Post Hoc Multiple Comparisons.

	Depression		Anxiety		Somatization	
	**(3) High**	**(2) Medium**	**(3) High**	**(2) Medium**	**(3) High**	**(2) Medium**
(2) Medium	(3) − (2) = 0.817<br>SP = 0.071<br>GHP = 0.119		(3) − (2) = 1.140<br>SP = 0.004<br>GHP = 0.013		(3) − (2) = 0.995<br>SP = 0.000<br>GHP = 0.004	
(1) Low	(3) − (1) = 1.635<br>SP = 0.000<br>GHP = 0.000	(2) − (1) = 0.818<br>SP = 0.001<br>GHP = 0.001	(3) − (1) = 2.187<br>SP = 0.000<br>GHP = 0.000	(2) − (1) = 1.047<br>SP = 0.000<br>GHP = 0.000	(3) − (1) = 1.800<br>SP = 0.000<br>GHP = 0.000	(2) − (1) = 0.805<br>SP = 0.000<br>GHP = 0.000

Note: SP=p-value from Scheffe test and GHP=p-value from Games-Howell test.

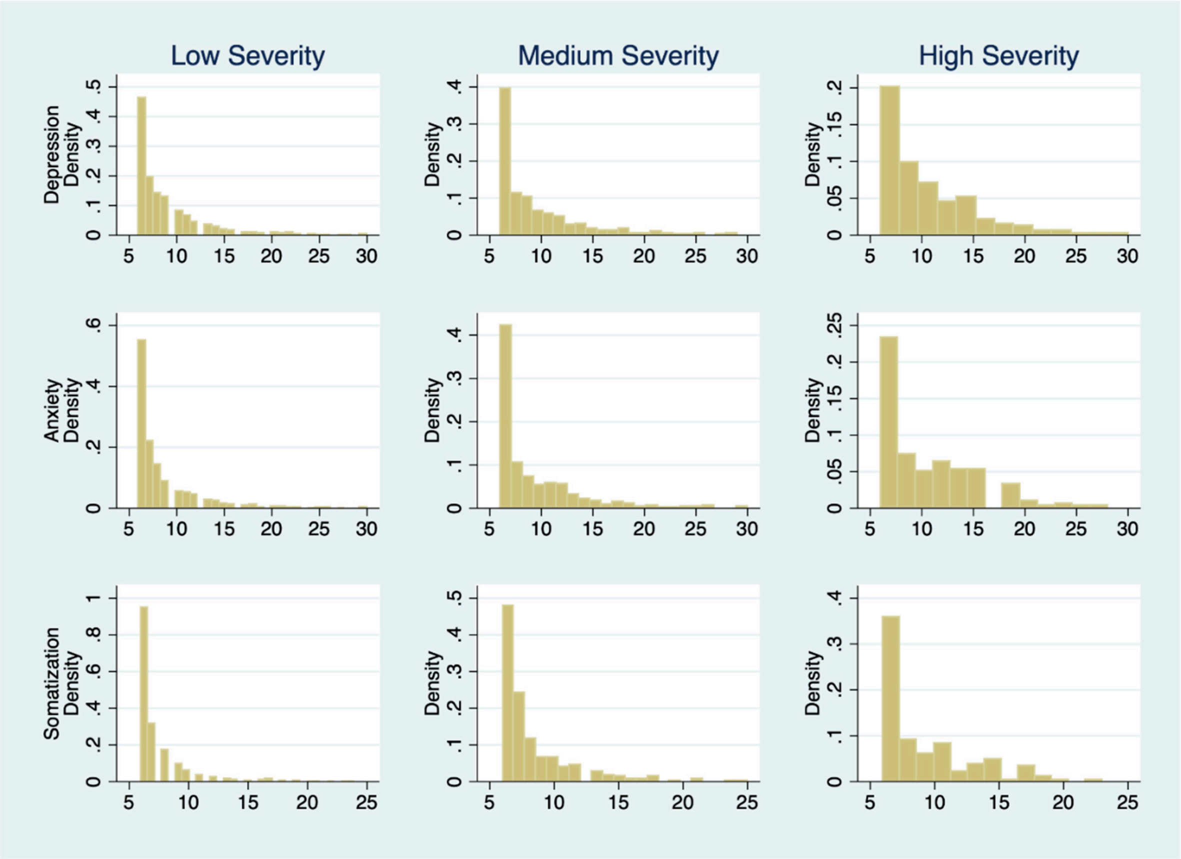

Supplementary Figure S1. Histograms of Mental Symptoms across Three Levels of Covid-19 Severity.

Cited References

1. Blanca, M. J., Alarcón, R., Arnau, J., Bono, R., & Bendayan, R. (2017). Non-normal data: Is ANOVA still a valid option? *Psicothema*, 29(4), 552-557. doi:10.7334/psicothema2016.383
2. Games, P., & Howell, J. (1976). Pairwise Multiple Comparison Procedures with Unequal N's and/or Variances: A Monte Carlo Study. *Journal of Educational Statistics*, 1(2), 113-125. doi:10.2307/1164979
3. Kruskal, W. H., & Wallis, W. A. (1952). Use of ranks in one-criterion variance analysis. *Journal of the American Statistical Association*, 47(260), 583-621. doi:10.1080/01621459.1952.10483441
4. Moder, K. (2016). How to keep the type I error rate in ANOVA if variances are heteroscedastic. *Österreichische Zeitschrift für Statistik*, 36(3), 179. doi:10.17713/ajs.v36i3.329
5. Welch, B. L. (1951). On the comparison of several mean values: An alternative approach. *Biometrika*, 38(3/4), 330-336. doi:10.2307/2332579

Supplementary 2. Natural Spline Procedures for Nonlinear Age Effect in Reference to Tables 4–7

Per the suggestion of Reviewer 1, we perform the procedures of natural spline in Stata to capture possible nonlinear effect of age on depression, anxiety, and somatization in ordinary least square (OLS) regression models. This operation is implemented by the *"mkspline"* command in Stata version 10.0 (StataCorp. 2015. *Stata 14 Base Reference Manual*. Stata Press: College Station, TX, USA. In Stata manual, the natural spline is documented as the "restricted cubic spline".).

We follow Harrell's recommendation (2001) to set 4 knots (k_1, k_2, k_3, and k_4) and generate 3 spline variables (X_1, X_2, and X_3) as the reparameterization of age. These spline variables are defined in the following way (Orsini and Greenland, 2011).

First, define quantile locations of 4 knots to be 5%, 35%, 65%, and 95% quantiles (Harrell, 2001) of the distribution of age. Given these quantiles, values of these four knots are k_1 =19, k_2 =24, k_3 =30, and k_4 =41, respectively.

Second, define $u_i = max(age - k_i\,, 0)^3$ with $i = 1,2,3,4$. For each knot i, if $age - k_i > 0$, then $u_i = (age - k_i)^3$; otherwise $u_i = 0$.

Third, given 4 knots we can define 3 spline variables for age (i.e. X_1, X_2, and X_3) using the formula:

$$X_1 = age$$

$$X_i = \frac{u_{i-1} - u_{m-1}\frac{k_m - k_{i-1}}{k_m - k_{m-1}} + u_m\frac{k_{m-1} - k_{i-1}}{k_m - k_{m-1}}}{(k_m - k_1)^2}$$

where $i = 2,3; m = 4$.

Supplementary Table S3 summarizes the definitions and values as discussed above.

Supplementary Table S3. Quantile Locations, Observed Values of Age, and Formulae of Spline Variables X_i at Each Knot k_i

**Knot #**	**Quantile location for k_i**	**Age at k_i**	**Spline variable X_i**
1	5%	19	$X_1 = age$
2	35%	24	$X_2 = \frac{u_1 - u_3\frac{k_4 - k_1}{k_4 - k_3} + u_4\frac{k_3 - k_1}{k_4 - k_3}}{(k_4 - k_1)^2}$
3	65%	30	$X_3 = \frac{u_2 - u_3\frac{k_4 - k_2}{k_4 - k_3} + u_4\frac{k_3 - k_2}{k_4 - k_3}}{(k_4 - k_1)^2}$
4	95%	41	

Finally, we estimate three sets of OLS regression models, one set for one mental health measure. Each set of OLS regression contains two models, which are defined as

Model a $y = a + b \times age + e$

Model b $y = a + b_1 \times X_1 + b_2 \times X_2 + b_3 \times X_3 + e$

In Model a, age enters the OLS regression as it is. In Model b, spline variables of age enter the OLS regression. After fitting each set of models, we plot predicted value of a mental health measure ($\hat{y}$) against age based on estimates from Model b. In addition, we display the 95% CIs of $\hat{y}$ at given age values (vertical bar), the non-linear spline fit from Model b (dash line), and the linear fit from Model a (solid line). Data for these plots are generated by the *"xblc"* user-written command in Stata (Orsini and Greenland, 2011).

Supplementary Figure S2 shows that for any mental health measure, linear and non-linear spline fits are very close. More importantly, the solid line of linear fit from Model a is almost always within the 95% CIs of $\hat{y}$ from Model b. Given these two pieces of evidence, we can conclude that these two

models are similar enough. And we prefer the more parsimonious Model a. In our main text, age enters OLS regression models as it is for each mental health measure.

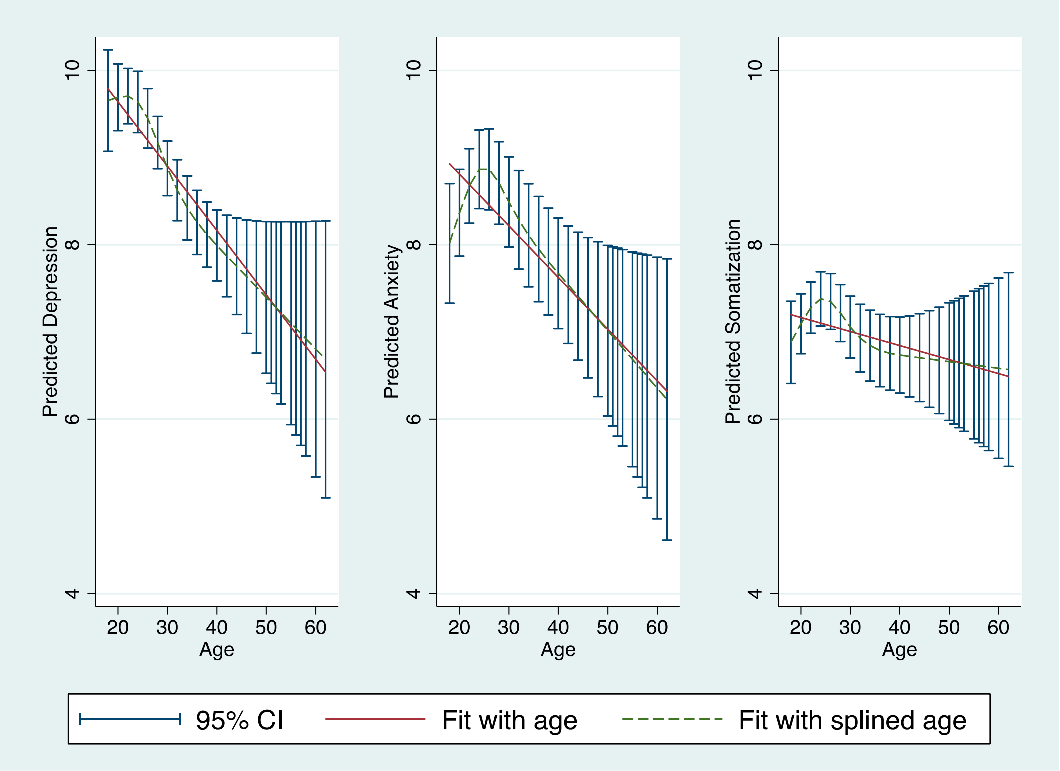

Supplementary Figure S2. Comparison of Model Fit for Two Different Ways of Incorporating Age to Ordinary Least Square Regressions.

To double-check this conclusion, we compare two full models (including interaction terms) of a mental health measure. These two models only differ in how to handle age, where Model a uses age and Model b uses spline variables of age. Supplementary Table S4 (next page) shows that no matter how we parameterize age, direction and significance of effects of our theoretical interests remain unchanged. Given this fact, we prefer the more parsimonious model setting and use age as it is in the main text.

Cited References

1 Harrell, F. E., Jr. (2001). *Regression Modeling Strategies: With Applications to Linear Models, Logistic Regression, and Survival Analysis*. New York: Springer.

2 Orsini, N., & Greenland, S. (2011). A procedure to tabulate and plot results after flexible modeling of a quantitative covariate. *The Stata Journal, 11*(1), 1-29. doi:10.1177/1536867x1101100101

Supplementary Table S4. OLS Regressions for Mental Symptoms with Different Handling of Age.

	Depression				Anxiety				Somatization			
	(1a)		(1b)		(2a)		(2b)		(3a)		(3b)	
	B/95% CI	*p*	B/95% CI	*p*	B/95% CI	*p*	B/95% CI	*p*	B/95% CI	*p*	B/95% CI	p
Gender	0.09 [−0.26, 0.45]	0.608	0.14 [−0.21, 0.50]	0.435	−0.19 [−0.55, 0.16]	0.293	−0.15 [−0.51, 0.21]	0.405	−0.12 [−0.36, 0.12]	0.332	−0.12 [−0.36, 0.12]	0.337
Age	−0.05 [−0.07, −0.02]	0.000			−0.05 [−0.08, −0.03]	0.000			−0.03 [−0.05, −0.01]	0.002		
X1			0.12 [−0.01, 0.26]	0.077			0.13 [−0.01, 0.27]	0.062			0.06 [−0.03, 0.16]	0.181
X2			−0.53 [−1.12, 0.06]	0.079			−0.64 [−1.24, −0.05]	0.033			−0.43 [−0.84, −0.03]	0.037
X3			1.07 [−0.37, 2.51]	0.146			1.39 [−0.05, 2.83]	0.059			1.04 [0.06, 2.03]	0.038
Marital status	0.21 [−0.23, 0.66]	0.349	0.19 [−0.26, 0.65]	0.401	0.63 [0.18, 1.08]	0.006	0.63 [0.18, 1.08]	0.006	0.47 [0.17, 0.78]	0.003	0.50 [0.20, 0.81]	0.001
Religious belief	0.39 [−0.09, 0.87]	0.114	0.39 [−0.09, 0.87]	0.113	0.64 [0.16, 1.12]	0.009	0.63 [0.15, 1.11]	0.010	0.91 [0.59, 1.24]	0.000	0.90 [0.57, 1.23]	0.000
Residence	0.14 [−0.21, 0.50]	0.430	0.18 [−0.18, 0.54]	0.325	0.06 [−0.30, 0.41]	0.756	0.10 [−0.26, 0.46]	0.591	−0.04 [−0.28, 0.21]	0.777	−0.01 [−0.25, 0.23]	0.937
CCP membership	0.09 [−0.36, 0.53]	0.701	0.10 [−0.35, 0.54]	0.669	−0.07 [−0.51, 0.38]	0.775	−0.06 [−0.50, 0.39]	0.797	0.08 [−0.23, 0.38]	0.622	0.07 [−0.23, 0.38]	0.636
Medium severity (vs. low)	0.82 [0.43, 1.21]	0.000	0.81 [0.43, 1.20]	0.000	0.96 [0.57, 1.35]	0.000	0.96 [0.57, 1.34]	0.000	0.65 [0.38, 0.91]	0.000	0.64 [0.38, 0.91]	0.000
High severity (vs. low)	1.66 [1.04, 2.27]	0.000	1.64 [1.03, 2.25]	0.000	2.03 [1.42, 2.64]	0.000	2.01 [1.40, 2.63]	0.000	1.58 [1.16, 2.00]	0.000	1.57 [1.15, 1.99]	0.000
SES	−0.15 [−0.37, 0.06]	0.168	−0.28 [−0.52, −0.05]	0.016	−0.02 [−0.24, 0.19]	0.828	−0.15 [−0.38, 0.09]	0.216	−0.06 [−0.20, 0.09]	0.453	−0.09 [−0.25, 0.07]	0.262
Health damaging behaviors	0.31 [0.13, 0.50]	0.001	0.28 [0.09, 0.46]	0.004	0.30 [0.11, 0.48]	0.002	0.27 [0.08, 0.45]	0.005	0.35 [0.22, 0.47]	0.000	0.34 [0.22, 0.47]	0.000
Health promoting behaviors	−0.41 [−0.58, −0.23]	0.000	−0.39 [−0.57, −0.22]	0.000	−0.15 [−0.33, 0.02]	0.083	−0.14 [−0.31, 0.03]	0.115	−0.06 [−0.18, 0.06]	0.330	−0.05 [−0.17, 0.07]	0.377
Values of individualism	−0.23 [−0.39, −0.06]	0.007	−0.22 [−0.38, −0.05]	0.010	−0.28 [−0.44, −0.12]	0.001	−0.27 [−0.43, −0.11]	0.001	−0.21 [−0.32, −0.09]	0.000	−0.20 [−0.32, −0.09]	0.000
Network intensity	−1.25 [−1.44, −1.06]	0.000	−1.26 [−1.45, −1.07]	0.000	−0.97 [−1.16, −0.78]	0.000	−0.98 [−1.17, −0.79]	0.000	−0.59 [−0.72, −0.46]	0.000	−0.59 [−0.72, −0.46]	0.000
Network extensity	−0.34 [−0.51, −0.18]	0.000	−0.34 [−0.50, −0.17]	0.000	−0.27 [−0.44, −0.11]	0.001	−0.27 [−0.43, −0.10]	0.002	−0.32 [−0.43, −0.20]	0.000	−0.31 [−0.43, −0.20]	0.000
SES × Medium severity	−0.28 [−0.68, 0.12]	0.169	−0.27 [−0.67, 0.13]	0.185	−0.24 [−0.64, 0.16]	0.241	−0.23 [−0.63, 0.17]	0.258	0.13 [−0.14, 0.40]	0.352	0.13 [−0.14, 0.40]	0.350
SES × High severity	−0.96 [−1.54, −0.38]	0.001	−0.93 [−1.50, −0.35]	0.002	−0.72 [−1.30, −0.14]	0.014	−0.69 [−1.27, −0.11]	0.019	−0.68 [−1.08, −0.29]	0.001	−0.67 [−1.07, −0.28]	0.001
Constant	9.82 [9.06, 10.59]	0.000	5.96 [2.92, 9.00]	0.000	9.41 [8.64, 10.17]	0.000	5.32 [2.29, 8.36]	0.001	7.75 [7.23, 8.27]	0.000	5.77 [3.70, 7.85]	0.000
Adjusted R^2	0.170		0.174		0.117		0.120		0.138		0.139	

N = 2015.

Supplementary 3. Procedures used to Generate Figure 2 Results

Each panel of Figure 2 shows the marginal effect of SES on one mental health measure at three different levels of Covid-19 severity. This Supplementary 3 summarizes steps to generate this figure.

Step 1. Estimate a linear OLS regression of one mental health measure (denoted as "y" in the following discussions) with all independent variables of our interest and the interaction between Covid-19 severity and SES:

$$y = a + \underbrace{b_1x_1 + b_2x_2 + \cdots + b_jx_j}_{Linear\ combination\ of\ j\ variables} + c_1Severity_{medium} + c_2Severity_{high} + c_3SES$$
$$+d_1Severity_{medium} \times SES + d_2Severity_{high} \times SES + e$$

Where a refers to constant, x_1 to x_j refer to all other independent variables except for two dummies of Covid-19 severity, SES, and interactions terms between them, b_1 to b_j refer to slopes of x_1 to x_j respectively, c_1 to c_3 are slopes for three main effects, d_1 and d_2 are slopes of two interaction terms, and e is the residual.

Step 2. Run the "*margins*" command of Stata 14.2 (StataCorp. 2015. *Stata 14 Base Reference Manual*. Stata Press: College Station, TX, USA) to generate three sets of predicted values of y, one set for a given level of severity. In each set, we select 11 values of SES (i.e. $k = -5, -4, -3, -2, -1, 0, 1, 2, 3, 4, 5$) to predict 11 values of y (denoted as $\hat{y}|SES = k$). In order to do so, we also need the estimated constant ($\hat{a}$), estimated slopes of $\hat{b}_1$ to $\hat{b}_j$, values of two severity dummies (their values are determined by the given level of severity), as well as sample means of $\bar{x}_1$ to $\bar{x}_j$. To simplify our expression, we let $\bar{X}\hat{b}$ to denote the value of the linear combination of $\hat{b}_1\bar{x}_1 + \hat{b}_2\bar{x}_2 + \cdots + \hat{b}_j\bar{x}_j$. Formulae to obtain values of $\hat{y}|SES = k$ are included in Supplementary Table S5.

Step 3. Draw a line plot of $\hat{y}|SES = k$ against SES to generate the first panel of Figure 2. Repeat these three steps for another two mental health measures to obtain panel 2 and panel 3 in Figure 2.

Supplementary Table S5. Values of Severity Dummies and Formulae to Predict Mental Health Measure Given Different Levels of Covid-19 Severity.

**Level of severity**	**Values of Dummies**		**Predicted values of dependent variable ($\hat{y}\|SES = k$)**
	$\boldsymbol{Severity_{medium}}$	$\boldsymbol{Severity_{high}}$	
Low	0	0	$= \hat{a} + \bar{X}\hat{b} + \hat{c}_3SES$
Medium	1	0	$= \hat{a} + \bar{X}\hat{b} + \hat{c}_1 + \hat{c}_3SES + \hat{d}_1SES$
Hight	0	1	$= \hat{a} + \bar{X}\hat{b} + \hat{c}_2 + \hat{c}_3SES + \hat{d}_2SES$
